# Detection of Chikungunya Virus RNA in Oral Fluid and Urine: An Alternative Approach to Diagnosis?

**DOI:** 10.3390/v16020235

**Published:** 2024-02-02

**Authors:** Leile Camila Jacob-Nascimento, Moyra M. Portilho, Rosângela O. Anjos, Patrícia S. S. Moreira, Christine Stauber, Scott C. Weaver, Uriel Kitron, Mitermayer G. Reis, Guilherme S. Ribeiro

**Affiliations:** 1 Instituto Gonçalo Moniz, Fundação Oswaldo Cruz, Salvador 40296-710, Brazil; biotecamila@gmail.com (L.C.J.-N.); moyra.portilho@hotmail.com (M.M.P.); rosaa.oliveira@hotmail.com (R.O.A.); patimoreira@live.com (P.S.S.M.); mitermayer.reis@fiocruz.br (M.G.R.); 2 Faculdade de Medicina da Bahia, Universidade Federal da Bahia, Salvador 40026-010, Brazil; 3 School of Public Health, Georgia State University, Atlanta, GA 30303, USA; cstauber@gsu.edu; 4 Department of Microbiology & Immunology and World Reference Center for Emerging Viruses and Arboviruses, University of Texas Medical Branch, Galveston, TX 77555, USA; sweaver@utmb.edu; 5 Department of Environmental Sciences, Emory University, Atlanta, GA 30322, USA; ukitron@emory.edu; 6 Yale School of Public Health, Yale University, New Haven, CT 06520-8034, USA

**Keywords:** chikungunya, RT-qPCR, molecular diagnosis, urine, oral fluid

## Abstract

To evaluate whether oral fluids (OF) and urine can serve as alternative, non-invasive samples to diagnose chikungunya virus (CHIKV) infection via RT-qPCR, we employed the same RNA extraction and RT-qPCR protocols on paired serum, OF and urine samples collected from 51 patients with chikungunya during the acute phase of the illness. Chikungunya patients were confirmed through RT-qPCR in acute-phase sera (N = 19), IgM seroconversion between acute- and convalescent-phase sera (N = 12), or IgM detection in acute-phase sera (N = 20). The controls included paired serum, OF and urine samples from patients with non-arbovirus acute febrile illness (N = 28) and RT-PCR-confirmed dengue (N = 16). Nine (47%) of the patients with positive RT-qPCR for CHIKV in sera and two (17%) of those with CHIKV infection confirmed solely via IgM seroconversion had OF positive for CHIKV in RT-qPCR. One (5%) patient with CHIKV infection confirmed via serum RT-qPCR was positive in the RT-qPCR performed on urine. None of the negative control group samples were positive. Although OF may serve as an alternative sample for diagnosing acute chikungunya in specific settings, a negative result cannot rule out an infection. Further research is needed to investigate whether OF and urine collected later in the disease course when serum becomes RT-qPCR-negative may be helpful in CHIKV diagnosis and surveillance, as well as to determine whether urine and OF pose any risk of CHIKV transmission.

## 1. Introduction

Chikungunya virus (CHIKV) is an alphavirus of the *Togaviridae* family. It was first detected near the border between Tanzania and Mozambique in 1952 [1,2]. In urban areas, *Aedes aegypti* and *Ae. albopictus* mosquitoes are the main vectors [3,4]. The autochthonous transmission of CHIKV in the Americas was first identified in the Caribbean region in 2013 [5]. Since then, CHIKV has spread across the American continents, causing multiple outbreaks and becoming a public health challenge in these regions of the world [6,7,8,9,10,11,12,13,14,15,16,17]. Brazil, where the first cases of chikungunya were detected in 2014, has been the most affected country in the Americas, with more than 1.6 million cases reported [16].

In humans, CHIKV infection can evolve asymptomatically or cause acute disease manifested by fever, headache, fatigue, myalgia, skin rash, joint swelling and especially arthralgia, which can be severe and last for months to years [14,18]. The diagnosis of chikungunya during its acute phase is based on clinical criteria, epidemiological data and, preferably, laboratory techniques. Reverse transcription–polymerase chain reaction (RT-PCR) is commonly used to detect CHIKV RNA in serum or plasma samples obtained up to seven days after the onset of symptoms [19,20,21]. Then, serological tests, such as enzyme-linked immunosorbent assay (ELISA), are the most frequently used to detect anti-CHIKV IgM antibodies seven days after the onset of symptoms or to detect antibody seroconversion between paired blood samples collected in the acute and convalescent phases of illness [21,22]. Other diagnostic approaches, such as viral isolation, hemagglutination inhibition assay and plaque reduction neutralization tests, are used less often and restricted mainly to research laboratories. Because CHIKV serological tests may cross-react with other alphaviruses, and most patients with chikungunya seek healthcare within the first three days after the onset of symptoms [23], serum RT-PCR is generally accepted as the gold-standard method for the definitive diagnosis of CHIKV infection.

However, obtaining serum from pediatric patients, especially neonates, can be complex. In these cases, the use of non-invasive biological samples is desirable. Testing easy-to-obtain samples would also facilitate the diagnostic investigation of febrile patients during periods of outbreaks when healthcare facilities are overcrowded or as part of the surveillance monitoring of virus transmission. Case reports [24,25] and small case series [26,27] have suggested that saliva and urine can offer viable means to detect CHIKV RNA through RT-PCR. However, only two main studies have compared the positivity rate of RT-PCR in the saliva, urine and serum of laboratory-confirmed chikungunya patients. These studies found superior serum performance (80.3% to 86.1% positivity) and a wide range of positivity in saliva (30% to 58.3%) and urine (8.3% to 23%) samples [28,29]. Because of the paucity of data on the potential usefulness of biological samples other than serum for diagnosing chikungunya, we investigated whether oral fluid (OF) and urine could serve as alternative specimens for diagnosing CHIKV infection via RT-qPCR.

## 2. Materials and Methods

### 2.1. Study Design and Samples Selection

To evaluate the performance of CHIKV RT-qPCR on OF and urine, we used samples obtained during a surveillance study aimed at detecting arbovirus infection among patients with acute febrile or exanthematous illness who were seeking healthcare at an emergency outpatient facility in Salvador, Brazil between September 2016 and March 2020. This surveillance study had the following inclusion criteria: age ≥ 6 months and self-reported skin rash or fever (or measured axillary temperature > 37.8 °C) lasting ≤ 7 days of duration at the time of consultation. After signing the informed consent form, or assent form when <18 years of age, the patients were interviewed to obtain demographic, epidemiological and clinical data and then underwent blood collection through venipuncture and OF collection with rayon-tipped swabs in the sublingual region. We also provided patients with a specimen collection cup and asked them to give a urine sample. To investigate arbovirus IgM antibody seroconversion, we invited the patients to return for blood collection during the convalescent phase (10–45 days after symptom onset). If the patient could not return, we offered to collect their blood samples at their homes. After collection, the blood samples were refrigerated at a constant 2–8 °C and transported under refrigeration on the same day to our laboratory at Fundação Oswaldo Cruz, where they were immediately centrifuged, aliquoted and frozen at −80 °C and −20 °C until molecular and serological testing, respectively. OF and urine underwent the same refrigeration and transportation protocol and were stored at −80 °C until molecular testing. The aliquots used in this evaluation were not thawed prior to the study.

The routine diagnostic work-up of arbovirus infection included testing the acute-phase sera of the enrolled participants using TRIOPLEX RT-qPCR-CDC, which detects CHIKV, dengue (DENV) and Zika virus (ZIKV) RNA [30], and using ELISA to investigate the presence of the DENV NS1 antigen (Dengue Early ELISA kit, Panbio Diagnostics, Brisbane, Australia). Both acute- and convalescent-phase sera were also assessed via ELISA for the detection of the anti-CHIKV IgM antibody (CHIKjj Detect™ IgM ELISA kits; InBios International, Seattle, WA, USA) and anti-DENV IgM antibody (Dengue IgM Capture ELISA, Panbio Diagnostics, Brisbane, Australia). OF and urine were not tested routinely. In Section 2.2, we describe in detail the RNA extraction and RT-qPCR protocols used to detect CHIKV RNA in acute-phase serum, OF and urine. 

We used the following criteria to select a stratified random sample (based on diagnostic criteria) of 51 patients with laboratory evidence of CHIKV infection and tested their OF and urine via RT-qPCR for comparison with results previously obtained in routine serum testing: (i) patients with CHIKV infection, as confirmed via RT-qPCR in the acute-phase sera (N = 19); (ii) patients with CHIKV IgM seroconversion between paired sera but with negative CHIKV RT-qPCR in the acute-phase sera (N = 12); and (iii) patients with CHIKV IgM detected in the acute-phase sera but with negative CHIKV RT-qPCR in the same sample (N = 20). As controls, we used OF and urine from a random sample of patients with non-arboviral diseases (NADs) (i.e., patients who were negative for arbovirus infection in the routine serum diagnostic investigation (N = 28)) and patients with RT-qPCR-confirmed dengue (N = 16). Control samples positive for DENV in RT-qPCR were also assessed via conventional RT-PCR for DENV serotype identification using oligonucleotides, as described by Lanciotti et al., 1992 [31]. Of the 16 patients in this group, 6 were DENV-1, 6 were DENV-2 and 4 were undetermined. The number of patients in each group was determined by the availability of paired serum, OF and urine; our goal was to have 20 patients per group. 

### 2.2. Detection of Chikungunya Virus RNA in Acute-Phase Serum, Oral Fluid and Urine

Viral RNA was extracted from the patients’ acute-phase samples (serum, OF and urine) using a Maxwell^®^ 16 Viral Total Nucleic Acid Purification Kit (Promega, Madison, WI, USA) according to the manufacturer’s specifications. For RNA extraction from the OF samples, we added 300 μL of phosphate-buffered saline solution to the tube containing the sublingual swab and vortexed it for 10 s. Then, we proceeded with RNA extraction as per the manufacturer’s instructions. The detection of CHIKV RNA in each sample was performed using TRIOPLEX RT-qPCR from the Centers for Disease Control and Prevention (CDC), which also detects DENV and ZIKV [30]. In this protocol, we added 10 μL of extracted viral RNA to 15 μL of the mix containing the solution from the SuperScript™ III Platinum™ One-Step RT-qPCR Kit (Invitrogen, Madison, WI, USA) and specific DENV, ZIKV and CHIKV primers and probes, generating a 25 μL reaction mix final volume that was amplified in a 7500 System (Applied Biosystems Inc, Waltham, MA, USA). The RT-qPCR program consisted of the first RT step of 30 min at 55 °C, then a 2 min denaturation step at 94 °C and, subsequently, 45 cycles of PCR steps (95 °C for 15 s, 60 °C for 1 min). We tested all samples in duplicate and the final cycle threshold (Ct) value was obtained as the average of the sum of the duplicate Ct values. Samples were considered positive if the Ct was <38. 

### 2.3. Statistical Analysis

All study data were entered into the digital database using the Research Electronic Data Capture (REDCap) software version 13.1.26 [32]. The patients’ demographic and clinical characteristics and the CHIKV RT-qPCR results were described by frequencies or medians and interquartile ranges (IQR) according to the patient’s study group. The data were analyzed using STATA software version 13 (StataCorp LP, College Station, TX, USA) and GraphPad Prism version 5.0 (GraphPad Software Inc., San Diego, CA, USA). Graphics were prepared using GraphPad Prism version 5.0 (GraphPad Software Inc., San Diego, CA, USA).

## 3. Results

### 3.1. Clinical Characteristics of Patients

The characteristics of the study patients and the results of the RT-qPCR in serum, OF and urine are shown in Table 1. The majority of the participants were female (between 50% and 69% of patients in each group) and the median age ranged from 24 to 40 years, which was lower in the control group of dengue patients and higher in the group of patients with CHIKV infection confirmed via RT-qPCR in serum. The three groups of patients with laboratory evidence of CHIKV infection and the control group of dengue patients were similar in the frequency of clinical signs and symptoms, although the group that was positive for CHIKV in RT-qPCR of serum tended to have slightly higher frequencies of symptoms typical of acute chikungunya, such as fever and arthralgia. In contrast, the group with anti-CHIKV IgM detected in the acute-phase sample had a higher frequency of rash and swollen joints, which may have been related to a later diagnosis, as was evidenced by the detection of IgM antibodies in the acute-phase sample and the higher IQR of the days of symptoms. Meanwhile, the group of patients with NAD had higher frequencies of a sore throat and cough. 

### 3.2. Chikungunya Virus RNA Detection in Oral Fluid and Urine

**Figure 1:**
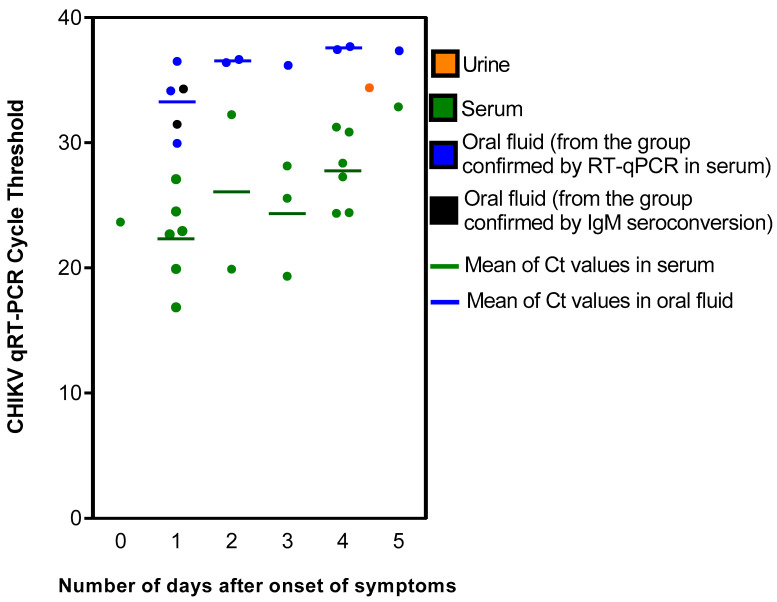
Chikungunya virus RT-qPCR cycle threshold (Ct) values in acute-phase serum, oral fluid and urine, according to the diagnostic criteria and the number of days between the onset of symptoms and sample collection.

Of the 19 patients with positive RT-qPCR for CHIKV in serum, 9 (47%) were also positive in RT-qPCR in the paired OF and 1 (5%) in the paired urine sample (Table 1 and Table 2). It was noteworthy that the CHIKV Ct values obtained in this group of patient samples were consistently lower in the serum samples compared to the paired OF and urine, suggesting lower viral loads in the latter samples (Table 1 and Table 2). This finding was consistent regardless of the number of days between the sample collection and the onset of symptoms (Figure 1). For both serum and OF, the greater the number of days between the sample collection and the beginning of symptoms, the higher the Ct levels obtained. In addition, in the nine patients with positive RT-qPCR for CHIKV in sera obtained between 0 and 2 days after the onset of symptoms, five (56%) had a positive RT-qPCR for CHIKV in OF. In contrast, for the ten patients with positive RT-qPCR for CHIKV in sera obtained between 3 and 5 days after the onset of symptoms, four (40%) had a positive RT-qPCR for CHIKV in OF.

Of the twelve patients with evidence of CHIKV infection detected only via CHIKV IgM seroconversion, two (17%) were positive for CHIKV by RT-qPCR in OF (Ct values > 31) and no patient was positive in the urine sample (Table 1 and Table 2). All 20 patients with evidence of CHIKV infection solely based on the detection of CHIKV IgM in acute-phase serum were negative for CHIKV in RT-qPCR of OF and urine. None of the samples from the two control groups were positive for CHIKV in RT-qPCR. 

## 4. Discussion

Our study on the utility of different biological samples for investigating CHIKV infection using RT-qPCR confirmed the findings of prior studies that serum should be the gold-standard sample used for diagnostic testing [28,29]. However, in this study, we found that 47% of the patients with acute chikungunya diagnosed via RT-qPCR in serum had detectable CHIKV RNA in OF (56% positivity when OF was collected within the first two days after symptom onset). Thus, in situations in which blood collection is not possible, OF can be used as an alternative sample, but a negative OF RT-qPCR result should not discount the likelihood of a CHIKV infection. In contrast, the frequency of which the urine samples were positive in RT-qPCR was too low (5%) to be useful, and this type of sample should not be used in CHIKV diagnosis. 

We also found that 17% of the patients with acute chikungunya confirmed solely through CHIKV IgM seroconversion had detectable CHIKV RNA in OF but not in urine. Thus, the frequency of RT-qPCR positivity in OF was approximately three times lower among chikungunya patients in the group with IgM seroconversion alone compared to that with the virus confirmed through serum RT-qPCR. This finding is not surprising, as patients with detectable CHIKV RNA in serum are more likely to also have detectable CHIKV RNA in other biological samples than patients without CHIKV RNA in serum. Nonetheless, we did detect CHIKV RNA in OF from serum RT-qPCR-negative patients, though the observed frequency was too low to guide routine testing of both serum and OF to increase diagnostic capacity. Further studies with increased numbers of chikungunya patients should be carried out to better assess any potential gain from testing paired serum and OF during the investigation of CHIKV infection. 

None of the OF or urine samples from the chikungunya patients confirmed only via CHIKV IgM in the acute-phase serum or from the control groups were RT-qPCR-positive. The failure to detect CHIKV RNA in OF and urine of patients with CHIKV infection confirmed only through the presence of CHIKV IgM in the acute-phase sample was in line with the low yield of RT-qPCR in OF and urine of patients who had a diagnosis of CHIKV infection solely based on IgM seroconversion. This finding indicates that the detection of CHIKV RNA in OF and urine is less likely when CHIKV RNA is not detected in serum. In addition, patients with negative RT-qPCR and positive IgM in the acute-phase serum are more likely to represent a group with longer disease duration compared to those who are RT-qPCR-positive or exhibit IgM seroconversion, as we observed in our study. This may also have hampered the detection of CHIKV RNA in OF and urine. 

Regardless of the chikungunya group to which the study patients belonged, samples from all patients with a positive RT-qPCR result from serum, OF or urine were collected within the first five days after the onset of symptoms. We found that the measured Ct values in serum and OF tended to increase from day 0 to day 5 after symptom onset, though a trend could not be evaluated in the urine samples because only one urine sample was positive. This finding was expected in serum samples, as Ct values in positive samples are inversely correlated with the viremia level, and CHIKV viremia declines over time [19,33]. However, a cohort study found the persistence of CHIKV RNA in the serum and saliva of patients for up to 60 days and in the urine for up to 95 days after the onset of the disease [29], indicating that molecular diagnosis may be attempted in samples collected during the post-acute phase of the illness. Because we tested only acute-phase samples, it was not possible to investigate the frequency of RT-qPCR positivity after the first week of symptom onset. Nevertheless, the findings of both the previously mentioned cohort study and our study were similar regarding increasing Ct values over time and lower Ct values in the serum samples than in the OF and urine samples. Combined, these results indicate that the detection of CHIKV RNA is more likely to occur in serum than in OF and urine samples and in samples collected in the acute phase than in samples collected in the post-acute or chronic phase of the illness.

Several reports have shown that CHIKV RNA can be detected in body fluids other than serum, such as saliva [26,28,29], urine [24,28,29,34], sperm [24,29], vaginal secretions [29], placenta or amniotic fluid [35], breast milk [36], synovial liquid [37] and cerebrospinal fluid [38,39]. Infectious CHIKV has also been detected in the saliva of mice, monkeys and humans [27], raising concerns about the potential for non-vector-borne transmission [40]. However, that viable and replicating viruses can be identified in saliva does not mean that the amount present can mediate direct person-to-person viral transmission. Additional studies are needed to elucidate whether transmission of CHIKV through non-vector-borne routes is indeed possible and to inform whether the presence of CHIKV genetic material in these body fluids can serve as a marker of the risk of direct transmission.

Our study has both limitations and strengths. This study, which included samples of 51 patients with laboratory evidence of CHIKV infection, is one of the most extensive in comparing the ability of RT-qPCR to detect CHIKV RNA in paired serum, OF and urine. However, the number of cases per group based on the confirmation criteria was small. Nonetheless, identifying and collecting paired biological samples from patients with chikungunya in the first week of symptoms is challenging because it is difficult to predict when and where a CHIKV outbreak will occur. Therefore, obtaining paired acute-phase samples from 51 patients with confirmed chikungunya can be considered a strength of our study. Furthermore, although the OF and urine samples tested did not show visual signs of blood, we cannot rule out the possibility of blood contamination due to a mucosal lesion. Nevertheless, none of the patients with CHIKV RNA detected in the OF samples reported gingival bleeding, and only one reported having oral ulcers. The only patient who had a positive urine sample did not report hematuria. In an additional limitation, the frequency of detection of CHIKV RNA in the patients’ acute-phase urine and OF samples, as well as the Ct of detection, may have been influenced by the chosen RNA extraction and RT-qPCR methods or by storing the samples at −80 °C instead of using fresh samples. However, the implementation of the same protocols for RNA extraction and RT-qPCR in all the tested samples ensured the consistency of our comparative study. Finally, in terms of strengths, our study differs from the other two main studies that investigated the usefulness of a set of biological samples for diagnosing CHIKV infection via RT-PCR [28,29] because it is the first to show that OF can be RT-qPCR-positive in patients without positive RT-qPCR in serum (but with IgM seroconversion). It also differs from the previous studies by including two control groups, one comprising patients with RT-PCR-confirmed dengue and one with non-arbovirus acute febrile illness, thus supporting the high specificity of CHIKV qRT-PCR in non-serum samples. 

In summary, our results confirm that serum is the best sample for RT-qPCR-based CHIKV diagnosis during acute disease, especially when collected in the first five days after the initial onset of symptoms. However, when serum cannot be obtained, or the laboratory detection of CHIKV is employed as part of surveillance efforts to monitor virus transmission trends among suspected patients, rather than for case diagnosis and management, testing OF may be attractive as a non-invasive alternative sample. However, while OF may prove helpful for surveillance or diagnosis in specific situations, a negative result should not be used to rule out a CHIKV infection. Our findings showed that the sensitivity of RT-qPCR performed in OF was about 50% that of the same assay performed in serum RT-qPCR. Nevertheless, because we found cases in which CHIKV RNA was detected in OF but not in serum, additional studies should be conducted to determine whether the parallel testing of serum and OF increases the capacity of case confirmation, to justify the routinization of the parallel testing of these two samples. 

## Figures and Tables

**Table 1:** Clinical, demographic and laboratory characteristics of the study patients.

Characteristic	Study Group				
	CHIKV Infection			Control Group	
	Detected via Serum RT-qPCR (N = 19)	Detected via IgM Seroconversion between Paired Samples (N = 12)	Detected via IgM in the Acute-Phase Sample (N = 20)	DENV Infection Detected via Serum RT-qPCR (N = 16)	Non-Arbovirus Diseases (N = 28)
	n/N (%) or median (interquartile range)				
**DEMOGRAPHICS**					
Sex (F)	10/19 (53%)	6/12 (50%)	13/20 (65%)	11/16 (69%)	14/28 (50%)
Age	40 (32–47)	32 (20–52)	35 (21–42.5)	24 (20–34)	31.5 (18.5–43)
Days after symptom onset	3 (1–4)	4 (1–5)	4 (3–6)	2.5 (1–3)	4 (3–7)
**CLINICAL MANIFESTATIONS**					
Fever	19/19 (100%)	11/12 (92%)	17/20 (85%)	16/16 (100%)	26/28 (93%)
Arthralgia	18/19 (95%)	9/12 (75%)	17/20 (85%)	12/16 (75%)	13/28 (46%)
Myalgia	18/19 (95%)	10/12 (83%)	17/20 (85%)	14/16 (88%)	15/28 (54%)
Headache	18/19 (95%)	9/12 (75%)	18/20 (90%)	14/16 (88%)	23/28 (82%)
Retro-orbital pain	12/19 (63%)	7/12 (58%)	10/20 (50%)	11/16 (69%)	8/28 (29%)
Conjunctival hyperemia	9/19 (47%)	3/12 (25%)	6/20 (30%)	10/16 (63%)	9/28 (32%)
Swollen joints	12/19 (63%)	5/12 (42%)	11/20 (55%)	2/16 (13%)	2/28 (7%)
Pruritus	10/19 (53%)	5/12 (45%)	15/20 (75%)	7/16 (44%)	8/28 (29%)
Rash	5/18 (28%)	4/12 (33%)	14/20 (70%)	5/16 (31%)	5/28 (18%)
Sore throat	7/19 (37%)	2/12 (17%)	6/20 (30%)	7/16 (44%)	12/28 (43%)
Cough	5/19 (26%)	4/12 (33%)	3/20 (15%)	5/16 (31%)	21/28 (75%)
Abdominal pain	5/19 (26%)	6/12 (50%)	7/20 (35%)	7/16 (44%)	15/28 (54%)
**CHIKV RT-qPCR RESULTS**					
** SERUM**					
Positive	19/19 (100%)	0/12 (0%)	0/20 (0%)	0/16 (0%)	0/28 (0%)
Cycle threshold	24.5 (22.7–28.3)	-	-	-	-
** ORAL FLUID**					
Positive	9/19 (47%)	2/12 (17%)	0/20 (0%)	0/16 (0%)	0/28 (0%)
Cycle threshold	36.6 (36.2–37.5)	32.9 (31.5–34.3)	-	-	-
** URINE**					
Positive	1/19 (5%)	0/12 (0%)	0/20 (0%)	0/16 (0%)	0/28 (0%)
Cycle threshold	34.4 (34.4–34.4)	-	-	-	-

**Table 2:** Values of RT-qPCR cycle threshold for detecting chikungunya virus RNA in positive serum, oral fluid or urine samples collected in the acute phase of infection.

Patient ID	Chikungunya Study Group Based on Diagnostic Method	Values of RT-qPCR Cycle Threshold		
		Serum	Oral Fluid	Urine
1	Serum RT-qPCR	32.3	36.4	34.4
2	Serum RT-qPCR	19.3	36.2	*
3	Serum RT-qPCR	27.1	36.5	*
4	Serum RT-qPCR	27.3	37.7	*
5	Serum RT-qPCR	22.7	34.2	*
6	Serum RT-qPCR	30.9	37.4	*
7	Serum RT-qPCR	19.9	36.6	*
8	Serum RT-qPCR	24.5	29.9	*
9	Serum RT-qPCR	32.9	37.3	*
10	IgM Seroconversion	*	31.5	*
11	IgM Seroconversion	*	34.3	*

* Negative.

## Data Availability

The data presented in this study are available on request from the corresponding author.
